# Comparison of the effectiveness of different scoring systems and biochemical markers in determining the severity and complications of acute pancreatitis

**DOI:** 10.55730/1300-0144.5989

**Published:** 2025-02-24

**Authors:** Ahmet Ali AKTAŞ, Pınar TAŞAR, Deniz SIĞIRLI, Sadık Ayhan KILIÇTURGAY

**Affiliations:** 1 Department of General Surgery, Faculty of Medicine, Bursa Uludağ University, Bursa, Turkiye; 2 Department of Biostatistics, Faculty of Medicine, Bursa Uludağ University, Bursa, Turkiye

**Keywords:** Acute pancreatitis, scoring systems, laboratory parameters, prognosis

## Abstract

**Background and study aim:**

The aim of the study is to demonstrate the effectiveness of different scoring systems and inflammatory markers in predicting the severity, local complications, pancreatic necrosis, and mortality of acute pancreatitis (AP).

**Materials and methods:**

The data of 357 patients whom the severity of pancreatitis was classified according to the Revised Atlanta Classification System diagnosed with AP were retrospectively examined. Also The APACHE II, BISAP, mCTSI, and Ranson scores of all patients were calculated. After determining the cut-off values for scoring systems and inflammatory markers with ROC analysis, comparison for AP severity, local complication, necrosis, and mortality.

**Results:**

In the study, 2.8% of patients had severe pancreatitis. It was found that the risk of developing local complications increased 2.82 times in cases with an 48^th^-h CRP value >192 mg/L compared to those below this threshold, and 48.96 times in cases with an mCTSI score >2 compared to ≤ 2 cases. It was found that having a Ranson score >4 increased the risk of mortality by 9.07 times compared to having a score of ≤4 (p = 0.038). It was observed that having a BISAP >2 increased the risk of severe AP by 11.79 times compared to ≤2. In cases where the 48^th^-h NLR value was >13.33, the risk of severe AP was found to have increased by 5.85 times.

**Conclusion:**

Although the superiority of scoring systems could not be demonstrated in our study, CRP and MCTSI for local complications, BISAP for severity and Ranson score for mortality were the determining variables.

## 1. Introduction

Acute pancreatitis is an inflammatory disease of the pancreas that develops as a result of the activation of digestive enzymes, which are normally inactive, in pancreatic acinar cells by various etiological factors.[1] The frequency of the disease is rapidly increasing, especially in European countries and the United States (USA) [2]. The clinical course of the disease has a wide spectrum, with 80% of cases being mild and self-limiting or 20% of cases being life-threatening severe disease. Mortality is less than 1% in mild disease, while in severe disease, mortality is at the level of 20%–40%.[3] Severe pancreatitis is associated with organ failure as well as local and systemic complications of the disease [4].

The ability of acute pancreatitis to present such differences in disease severity in its clinical course has led clinicians from the 1970s to the present day to develop scoring systems that can predict prognosis and complications closely related to prognosis using certain parameters. Indeed, being able to predict severe disease at presentation will allow for close monitoring of the patient, aggressive resuscitation, and referral to a specialized center if necessary. In this regard, there are many developed scoring systems [5].

Ranson Criteria, Acute Physiology and Chronic Health Enquiry (APACHE II), BISAP (Bedside Index Of Severity In Acute Pancreatitis) score, mCTSI (Modified Computed Tomography Severity Index) score, Revised Atlanta Criteria, Determinant Based Classification (DBC) are currently in clinical use and their prognostic values are being studied. The Ranson score is criticized for being impractical due to its inclusion of 11 variables and requiring a 48-h period. The APACHE II system is a system that includes many variables, some of which are not always accessible, and are relatively difficult to calculate. The mCTSI score is based on morphological findings and these findings emerge over time. Therefore, its use in the early period can pose disadvantages in terms of both a decrease in diagnostic value and an increase in cost. The BISAP score appears to be a successful system due to its fewer parameters, being memorable, easy to calculate, and as successful as other systems.

In addition, in acute pancreatitis, inflammatory markers such as C-Reactive Protein (CRP) and procalcitonin; although not included in the scoring systems, their relationships with important prognostic markers such as organ failure and infected necrosis have been examined in many studies [6].

There is no consensus on the superiority of a single scoring system in determining the course and prognosis of the disease. In this study, it is aimed to determine the scoring systems and inflammatory markers that show optimal correlation with the results associated with the disease by examining the file records of patients who were hospitalized and treated with a diagnosis of acute pancreatitis in our center during the 5-year period between 2018–2022.

## 2. Methods

Approval was obtained from the Clinical Research Ethics Committee of Bursa Uludağ University Faculty of Medicine on January 17, 2023, with the decision number 2023-2/11. During the study process, the principles of the Helsinki Declaration were adhered to. The study is a retrospective study aimed at investigating the effectiveness of some scoring systems and inflammatory parameters in predicting the severity of the disease and complications in patients who applied to the emergency department and outpatient clinics of Bursa Uludağ University Hospital, a tertiary center, between January 2018 and December 2022, and were hospitalized with a diagnosis of acute pancreatitis according to the Atlanta criteria.

The diagnosis of acute pancreatitis was made when at least two of the three criteria specified in the Revised Atlanta Classification were met: 1. Abdominal pain that is prominent in the epigastric region and radiates to the back, 2. Serum amylase/lipase level being at least 3 times higher than the upper limit of normal, 3. Detection of pancreatic inflammation findings with imaging methods (computed tomography and magnetic resonance).

The demographic data of the patients, vital parameters recorded at the time of application and treatment process, laboratory values, imaging results, service, intensive care and total hospitalization durations, interventions made during the hospitalization process, morbidity, and mortality were obtained from the digital record archive. The laboratory parameters required for the scoring systems were obtained from the blood test results at admission. The 48-h laboratory values required for the Ranson score were also recorded. Radiological images (CT and Chest X-rays) were examined for BISAP and mCTSI. With these obtained data, the scores of Ranson, BISAP, APACHE II, mCTSI, Revised Atlanta and Determinant Based Classification cathegories of the patients were obtained. In addition, some inflammatory markers such as CRP, procalcitonin, NLR, LMR, PLR values at the time of application, 48th and 72nd h of hospitalization were also recorded.

The severity of the disease was classified according to the Revised Atlanta Classification, and the effectiveness of the scoring systems in determining mild, moderate, and severe acute pancreatitis was examined according to this classification. Organ failure findings were determined according to the Modified Marshall scoring system. Patients who had at least 48 h of hospitalization were included in the evaluation as it was necessary to determine the severity of the disease.

Patients under the age of 18, those with active hematological malignancy or solid tumor, those with active pregnancy or in the postpartum period, those with known chronic pancreatitis or chronic pancreatitis findings in their imaging, those who had an attack of acute pancreatitis in the last 90 days, those whose data could not be reached or insufficient data were detected were excluded from the study.

According to the Revised Atlanta Classification, groups of mild, moderate, and severe acute pancreatitis were determined, and the predictive values of Ranson, BISAP, APACHE II, mCTSI scores in determining the severity of the disease were examined separately. The CRP, procalcitonin, NLR, LMR, PLR values of the patients at the time of application, 48th and 72nd h were calculated, and the predictive values of these inflammatory markers and the scores obtained in terms of important morbidity and mortality markers such as mortality, hospitalization duration, local complications, necrosis, and necessity of intervention were also examined separately.

### 2.1. Statistical analysis

All statistical analyses were performed using IBM SPSS for Windows 28.0.0.0 (IBM SPSS, Chicago, IL, United States) and MedCalc Statistical Software version 22.009. Numerical variables are presented as median (minimum–maximum) due to their nonnormal distribution, and categorical variables are presented as n (%) values. The Mann–Whitney U test was used to compare numerical variables between two independent groups. Pearson’s chi-square and Fisher’s exact chi-square tests were used to compare categorical variables between groups. Relationships between numerical variables were examined using Spearman’s rank correlation coefficient. ROC analysis was performed to reveal the diagnostic performances of Ranson, BISAP, APACHE II, mCTSI scoring systems and laboratory parameters in determining various endpoints, and sensitivity, specificity, positive predictive value (PPV) and negative predictive values (NPV) were estimated, and optimal cut-off values were determined according to Youden’s J index. Logistic regression analysis was performed by including systems and markers that gave significant AUC values in the ROC analysis to determine risk factors for mortality, local complications, necrosis, and disease severity. In all statistical tests, p < 0.05 was considered significant.

## 3. Results

The study included 357 cases of acute pancreatitis. Of these cases, 164 (45.9%) were male and 193 (54.1%) were female. The median age was found to be 58 (19–91). 163 (45.7%) of the patients had no comorbidities. At least one or more comorbidities were observed in 194 (54.3%) patients, with the most common comorbidities being hypertension in 139 (39.2%) patients, diabetes mellitus in 74 (20.8%) patients, and coronary artery disease in 74 (20.8%) patients.

When the etiology of pancreatitis was examined, it was found that 259 (76.6%) of the patients had biliary causes, 27 (8%) were post-ERCP, 25 (7.4%) had hyperlipidemia, 12 (3.6%) were due to alcohol, and 34 were due to other causes.

When patients were categorized according to the Revised Atlanta criteria for the severity of pancreatitis, it was found that 273 (76.5%) patients had mild pancreatitis, 74 (20.7%) patients had moderate pancreatitis, and 10 patients (2.8%) were in the severe pancreatitis group. Severe pancreatitis was seen in 3.1% of male patients, while this rate was 2.6% in females.

Intensive care admission occurred in 18 (5%) of the patients. The total hospital stay was a median of 7 (2–140) days, the ward stay was a median of 7 (0–140) days, and the intensive care stay was a median of 1 (0–87) day. While 70% of the 10 patients followed with severe pancreatitis needed intensive care, this rate was 13.5% in patients with moderate pancreatitis and 0.4% in patients with mild pancreatitis. This difference was found to be statistically significant (p < 0.001).

Among the 257 patients found as a result of CT imaging, local complications of acute pancreatitis were not detected in 192 (74.7%) cases, while acute peripancreatic fluid collection was the most common complication with 32 (12.5%) cases. Acute necrotic collection findings were present in 28 (10.9%) patients. Among those who developed local complications, 89.1% were patients with moderate severity pancreatitis. The rate of local complication occurrence was significantly higher both from patients with severe pancreatitis (7.8%) and from patients with mild pancreatitis (3.1%) (p < 0.001).

Endoscopic, percutaneous, or surgical intervention was applied to a total of 52 (14.6%) patients. Of these, radiological drainage was performed on 23 patients and endoscopic retroperitoneal drainage was performed on 32 patients. While there was a need for intervention in 40% of patients with severe pancreatitis, this rate constituted 29% of patients with moderate pancreatitis and 9.9% of mild AP cases. Patients with severe pancreatitis had a similar need for intervention with patients with moderate pancreatitis (p = 0.48), it was significantly higher than mild pancreatitis cases (p = 0.01).

Pancreatic necrosis was present in 42 of the patients and it was detected in some patients through MRI imaging, which was performed to determine the etiology, in addition to CT imaging. Of the cases with necrosis, 69% were patients with moderately severe pancreatitis. Patients with moderate severity pancreatitis accounted for 69% of the cases where necrosis was observed. The rate of necrosis occurrence was significantly higher both from patients with severe pancreatitis and from patients with mild pancreatitis (p < 0.001).

Mortality was seen in 10 patients, 7 of whom were severe pancreatitis cases. The rate of mortality occurrence in patients with severe pancreatitis was significantly higher than both groups (p < 0.001).

### 3.1. Disease severity

**Figure 1:**
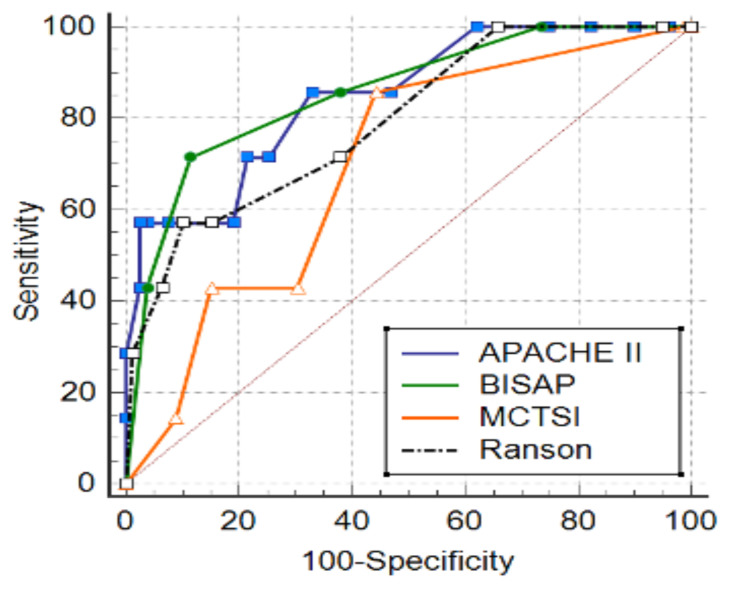
ROC curves showing the diagnostic performance of scoring systems in relation to AP severity.

The cut-off and AUC values of the scoring systems can be seen in Table. When the performance of the scoring systems in distinguishing between mild-moderate and severe pancreatitis cases was examined, the highest AUC value of 0.884 was obtained with a cut-off value of >2 for the BISAP score (p < 0.0001) (Figure 1). The AUC value is statistically significant in distinguishing AP severity (p = 0.0002). At these cut-off values, the Ranson score had the highest specificity, while the Apache II score had the highest sensitivity.

In distinguishing the severity of AP, the CRP level had low AUC values at 24th h (AUC 0.610, p = 0.3), 48th h (AUC 0.658, p = 0.13), and 72nd h (AUC 0.608, p = 0.40). When procalcitonin levels were examined in this regard, it had a low AUC value at 24th h. At 48th h, procalcitonin values had a cut-off of >0.92 ng/mL and a high AUC value (0.951) was obtained (p < 0.0001). Similarly, the 72nd h procalcitonin had a cut-off of >6.18 ng/mL and an AUC value of 0.971 (p < 0.0001).

The NLR (24th h) had a cut-off of >10.35, with an AUC value of 0.757 (p = 0.01). The calculated NLR at 48th h had a cut-off of >13.33 with an AUC value of 0.893 (p < 0.0001). The 72nd h NLR had a cut-off of >10.30 with an AUC value of 0.843 (p < 0.0001). However, LMR and PLR values did not show significant diagnostic performance in distinguishing AP severity at 24th, 48th, and 72nd h.

### 3.2. Local complications

**Figure 2:**
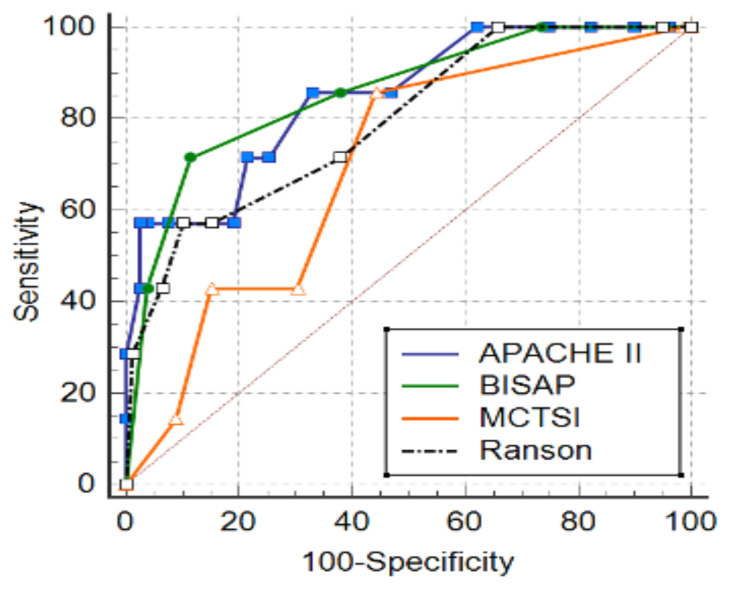
ROC curves showing the diagnostic performance of scoring systems in relation to local complications.

In predicting local complications in AP, the mCTSI had the best AUC value. Similarly, mCTSI had the highest specificity and sensitivity among the scoring systems. The cut-off for mCTSI in predicting local complications was found to be >2. Among other scoring systems, APACHE II and BISAP scores had low AUC values in predicting local complications of AP (Figure 2). For the Ranson score, a cut-off value of >2 and a low AUC value (0.638) showed significant diagnostic performance (p < 0.01).

In predicting local complications, the 24^th^-h CRP had a cut-off value of 142 mg/L and a low AUC value of 0.586. In contrast, the 48^th^-h CRP had a cut-off value of >192 mg/L and an AUC of 0.704 (p < 0.01), and the 72^nd^-h cut-off value was 141 mg/L with an AUC of 0.700 (p < 0.01). When looking at procalcitonin levels as another inflammatory marker, the 24^th^-h value had a low AUC of 0.564, the 48^th^-h value had an AUC of 0.609, and the 72^nd^-h value had a cut-off of >0.26 ng/mL and an AUC of 0.648 (p = 0.03).

The Neutrophil/Lymphocyte Ratio (NLR) had an AUC of 0.586 at 24^th^-h (p = 0.057), a cut-off of >8.913 with an AUC of 0.671 at 48^th^-h (p < 0.0001), and a cut-off of >6.9556 with an AUC of 0.718 at 72^nd^-h (p < 0.0001). The Platelet/Lymphocyte Ratio (PLR) at 24^th^, 48^th^, and 72^nd^ h had low AUC values and were statistically insignificant. Similarly, the LMR values at 24^th^, 48^th^, and 72^nd^ h had low AUC values.

### 3.3. Necrosis

In the ROC analyses for scoring systems to predict pancreatic necrosis in AP, the mCTSI score had a cut-off value of >4 and the highest AUC value (0.923) was obtained (p < 0.0001). The Ranson score had significant diagnostic performance in predicting pancreatic necrosis with a cut-off of >2 and an AUC value of 0.698 (p < 0.0001). In contrast, the APACHE II score had an AUC value of 0.653 (p < 0.0001) and the BISAP score had an AUC value of 0.605 (p = 0.02).

For the inflammatory markers, the 24^th^-h CRP had an AUC value of 0.645 (p = 0.46), the 48^th^-h CRP had an AUC value of 0.670 (p < 0.0001), and the 72^nd^-h CRP level had a cut-off value of >140.7 mg/L and an AUC value of 0.741 (p < 0.0001).

Similarly, the PLR values at admission, 48^th^ and 72^nd^ h also revealed lower AUC values (AUC and p-values respectively, for PLR24: 0.591, p = 0.06, for PLR48: 0.567, p = 0.14, for PLR72: 0.572, p = 0.16).

The NLR at 24^th^-h had a low AUC value of 0.615 (p = 0.02). At 48^th^-h, an AUC value of 0.689 (p < 0.0001) was obtained, and at 72^nd^-h, a cut-off of >8.7965 and an AUC value of 0.733 were obtained (p < 0.0001). For LMR48, a cut-off of ≤1.5718 and an AUC of 0.735 were obtained (p < 0.0001), and for LMR72, a cut-off of ≤1.4663 and an AUC of 0.727 were obtained (p < 0.0001). For PLR24, an AUC of 0.591 was obtained (p = 0.06), for PLR48, an AUC value of 0.567 was obtained (p = 0.14), and for PLR72, a low AUC value of 0.572 was obtained (p = 0.16).

### 3.4. Mortality

**Figure 3:**
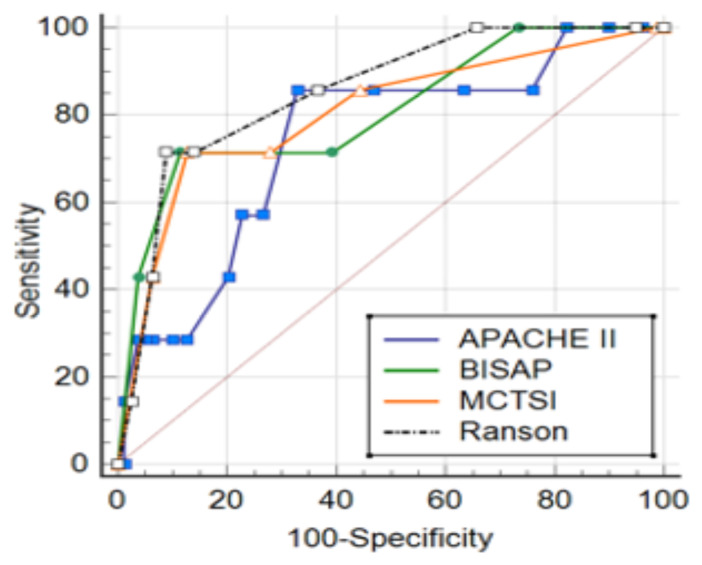
ROC curves showing the diagnostic performance of scoring systems in relation to mortality.

In the ROC analysis conducted to evaluate the diagnostic performance of the scoring systems in relation to mortality, the Ranson score had a cut-off value of >4 and had the highest AUC value (0.918) (p < 0.0001). It had the highest specificity at this cut-off value. For the APACHE II score, a cut-off value of >7 was obtained, for the BISAP score, a cut-off value of >2 was obtained, and for the mCTSI score, a cut-off value of >4 was obtained (p < 0.0001) (Figure 3).

For the inflammatory markers, the 24^th^-h CRP had an AUC value of 0.614 (p = 0.31), the 48^th^-h CRP level had a cut-off of >197.5 mg/L and an AUC value of 0.807 (p < 0.0001), and the 72^nd^-h CRP level had a cut-off of >222.2 mg/L and an AUC value of 0.808 (p = 0.0006). For the 48-h procalcitonin, a cut-off of >9.75 ng/mL and an AUC value of 0.992 were obtained (p < 0.0001), and for the 72^nd^-h procalcitonin, a cut-off of >6.18 and an AUC value of 0.956 were obtained (p < 0.0001).

For the NLR24, a cut-off of >10.35 and an AUC value of 0.834 were obtained (p < 0.0001), and for the NLR48, a cut-off of >9.65 and an AUC value of 0.918 were obtained (p < 0.0001). For the LMR and PLR, low AUC values were obtained at 24th, 48th, and 72nd h.

### 3. 5. Comparison of biomarkers and scoring systems in predicting severity, local complications, necrosis, and mortality

After determining the cut-off values that show significant discriminative performance for scoring systems and inflammatory markers with ROC analyses, logistic regression models have been created for AP severity, local complication, necrosis, and mortality.

In predicting AP severity, in the stepwise backward logistic regression analysis performed including the NLR values at 24^th^, 48^th^, and 72^nd^ h, in addition to the APACHE II, BISAP, Ranson, and mCTSI scoring systems which provide the best and significant AUC values, the final model retained the BISAP score and the 48^th^-h NLR values as significant variables (Omnibus test p < 0.001 for the model). It was observed that having a BISAP >2 increased the risk of severe AP by 11.79 times compared to ≤2. In cases where the 48^th^-h NLR value was >13.33, the risk of severe AP was found to have increased by 5.85 times.

In predicting local complications, the final model in the stepwise backward logistic regression analysis, which included the Ranson, BISAP, and mCTSI scores that provided the best and significant AUC values, as well as the 48^th^ and 72^nd^ h CRP values and the 72^nd^ h NLR value, retained the mCTSI score and the 48^th^ h CRP level (Omnibus test p < 0.001 for the model). It was found that cases with an mCTSI score of >2 had a 48.96 times higher risk of developing local complications compared to cases with ≤2 (p < 0.001). In cases where the 48^th^ h CRP value was >192 mg/L, the risk of developing local complications was found to be 2.82 times higher than in cases below this threshold (p = 0.03).

In distinguishing pancreatic necrosis, the final model in the stepwise backward logistic regression analysis, which included the Ranson, BISAP, APACHE II, and mCTSI scores that provided the best and significant AUC values, as well as the 48^th^ and 72^nd^ h LMR values and the 72^nd^ h NLR and CRP values, retained the mCTSI score, the APACHE II score, and the 48^th^ h LMR ratio (Omnibus test p < 0.001 for the model). The APACHE II score was not found to be significant for necrosis (p = 0.07). It was found that patients with an LMR ratio of >1.57 had a 7.94 times increased risk of developing pancreatic necrosis compared to patients with ≤1.57 (p = 0.007). In patients with an mCTSI score of >4, the risk of pancreatic necrosis was found to be 25.51 times higher compared to patients with ≤4 (p < 0.001).

In the stepwise backward logistic regression analysis performed by including BISAP, APACHE II, Ranson, and mCTSI into the model for mortality, only the variables of BISAP and Ranson scores remained in the final model (Omnibus test p < 0.001 for the model). While the BISAP score was not significant (p = 0.100), it was found that having a Ranson score >4 increased the risk of mortality by 9.07 times compared to having a score of ≤4 (p = 0.038).

## 4. Discussion

Acute pancreatitis is a clinical picture caused by the local and systemic response developed in the organism as a result of inflammatory pancreatic damage triggered by various etiological factors. This picture can vary from a self-limiting mild disease to a severe disease resulting in multiple organ failure and mortality. Especially in the early stages of the disease, it is extremely important to catch the clues of the severe clinical picture and determine the management strategy accordingly to reduce mortality and for the effective use of patient care facilities.

In acute pancreatitis (AP), the severity of the disease is often classified in the literature as mild and severe, which is different from the categorization of mild-moderate and severe as stated in the Revised Atlanta Criteria. One reason for this is the establishment of strict criteria such as pancreatic necrosis, local complications, sepsis, and the necessity of surgical intervention as determinants of disease severity before the consensus of the Revised Atlanta Criteria [7]. A second reason could be the difficulty in distinguishing between moderate AP and severe AP cases and the inadequacy of the current scoring systems in this regard [8]. At this point, the distinction between moderate and severe AP is determined by improving or persistent organ failure, and in studies where cases are reported as severe AP, there is a high probability that moderate and severe AP cases are intertwined. In studies reporting large case series in this way, it is expected that 15%–25% of cases will follow a severe pancreatitis course. Looking at our study in this direction, it is seen that in accordance with the literature, 77.6% of all cases are mild AP and 22.4% are in the moderate and severe AP group according to the disease severity categories of the Revised Atlanta Classification.

In acute pancreatitis, the expected overall mortality rate has been reported as 3%–4% [9]. In our study, the mortality rate was 2.8%. The reason for the lower mortality rate compared to the literature is that severe pancreatitis patients in our study constitute a small part of the cases (2.8%). The expected mortality rates according to disease severity are 0%–1% for mild AP and 15%–35% for severe AP cases [10]. When considering the classification in the Revised Atlanta Criteria, the mortality rate in the literature varies between 15%–20% for moderate or severe pancreatitis [11]. In our study, the mortality rate in moderate and severe AP cases is in line with the literature at 12.7%. It is observed that 18 patients required intensive care admission. This indicates that some patients in the moderate-severity pancreatitis group also required intensive care.

In our study, local complications were quite rare in mild AP cases (1.1%), while local complications were frequently encountered, especially in moderate (89.1%) and severe AP (55.6%) cases. This situation is highly likely related to the fact that the main determinant in moderate AP cases is local complications, and in severe AP cases, the main determinant is persistent organ failure, as emphasized in the Revised Atlanta Criteria [12]. In 10.9% of the cases in our study, acute necrotic collection was observed from local complications. In the literature, necrosis is observed in 10%–15% of acute pancreatitis cases [13].

In predicting local complications (>2 cut-off) and necrosis (>4 cut-off), the mCTSI scoring system had the highest AUC value and significant diagnostic performance among our study. It had the highest sensitivity, specificity, and negative predictive value. In a study comparing scoring systems in the literature, a cut-off value of ≥4 was found for mCTSI, and it was shown to have the highest AUC value and sensitivity in the analysis for local complications [14]. The standout success of the mCTSI scoring system is likely due to the parameters that make up the system being directly local AP findings, resulting in a more predictable outcome. In our study, while the Ranson score was statistically significant, it had a low AUC value, sensitivity, and specificity. Therefore, in our study, the Ranson score, like the APACHE II and BISAP scoring systems, had insignificant diagnostic performance in predicting local complications and necrosis that could develop during the AP process.

In laboratory tests, it was observed that the CRP and NLR values measured at 48^th^ and 72^nd^ h yielded successful results. The values obtained for both laboratory parameters at the 72^nd^ h had a higher AUC value and higher sensitivity compared to 48^th^ h. In our study, we could not find a successful laboratory parameter that could predict local complications at the time of admission. Although different cut-off values are reported for both CRP and NLR in the literature, in a recent meta-analysis [15] that included 181 studies, when studies with a cut-off value of >150 mg/L for CRP value at admission and at 48^th^ h were examined, it was found that the CRP value at 48^th^ h yielded more sensitive and specific results with better AUC values.

Similarly, in predicting pancreatic necrosis, CRP values measured within the first 24 h cannot provide sufficient diagnostic performance, while values measured from 48^th^ h onwards can demonstrate significant performance in this regard. This chronological delay has been reported to be due to the natural process of CRP synthesis as a result of cytokine stimulation [16]. In our study as well, it was found that the CRP value could only show significant predictive performance for both pancreatic necrosis and other local complications from 48^th^ h onwards.

When examining scoring systems that provide effective predictive data for mortality and disease severity in Acute Pancreatitis (AP), it was found that all of the Ranson, APACHE II, BISAP, and mCTSI systems showed statistically significant performance. However, the highest AUC value (severity: 0.881, mortality: 0.918) belonged to the Ranson scoring system. For mortality, while the Apache II score had the highest sensitivity with a >7 cut-off, the highest specificity was in patients with a Ranson score above >4. Both patients with a BISAP score >2 and a Ranson score >4 had the highest specificity in predicting AP severity and mortality. It was also observed in our study that the Apache II score is not specific for pancreatitis, but it is an important scoring system in determining mortality in other diseases, especially in intensive care patients.

In mortality, in the stepwise backward logistic regression analysis performed by including BISAP, APACHE II, Ranson, and mCTSI into the model, only the variables of BISAP and Ranson scores remained in the final model (Omnibus test for the model: p < 0.001). While the BISAP score was not significant (p = 0.100), it was found that having a Ranson score >4 increased the risk of mortality by 9.07 times compared to having a score of ≤4 (p = 0.038). In a published meta-analysis [17], a cut-off value of >2 was found for the BISAP score and at this cut-off value, while the BISAP score had high specificity and low sensitivity for mortality, it was found that having an Apache II score >7 had low specificity and high sensitivity. In the same meta-analysis, the Ranson score achieved the highest AUC value in mortality with a >2 cut-off value. For severity assessment, while >2 cut-off values had a high AUC value, BISAP had the highest specificity, and the APACHE II score had the highest sensitivity. In our study, similar data and cut-off values to this meta-analysis were obtained. In predicting severity, only BISAP remained as a significant variable in the final model (Omnibus test for the model: p < 0.001). It was found that having a BISAP score >2 increased the risk of severe AP by 19.44 times compared to having a score of ≤2 (p = 0.001).

In a review published by Ong et al. [18], it was emphasized that despite the Ranson score being older than the APACHE II, BISAP, and mCTSI scoring systems, it has similar success in predicting both mortality and disease severity. In recent studies related to the Ranson score [14, 19, 20], it has been stated that it demonstrates performance close to or superior to other scoring systems, and its specificity is higher than these scoring systems. Gao et al. [17] reported that the Ranson score provides higher AUC values for mortality compared to the APACHE II and BISAP scoring systems. In our study, in line with this data, the Ranson score provided the best AUC value for mortality, and the specificity of the Ranson score was found to be higher in mortality and disease severity.

On the other hand, it is also stated in some recent significant prospective studies that the BISAP and APACHE II scoring systems have demonstrated more successful performance [21–23].

When looking at the laboratory parameters, the NLR at admission and at 48^th^ h, and the procalcitonin levels at 48^th^ and 72^nd^ h showed significant diagnostic performance in terms of mortality and disease severity. Especially, the procalcitonin value at 48^th^ h had the highest AUC, specificity, and sensitivity for mortality, while the procalcitonin values at 72^nd^ h were more significant for severity.

In a study conducted by Liu et al. [24], it was found that the NLR demonstrated a performance as successful as the BISAP score. However, Binnetoğlu et al. [25] have pointed out that commonly used antibiotic agents in AP treatment could affect cell counts such as neutrophils and lymphocytes, and therefore the prognostic value of the NLR in AP is controversial. Although the NLR value was found to be significant in terms of prognosis in our study, the fact that patients given antibiotic therapy were not excluded requires consideration on the results obtained.

In our study, serum procalcitonin levels at 48^th^ and 72^nd^ h showed statistically significant diagnostic performance in predicting both mortality and disease severity. In a prospective study comparing procalcitonin levels with widely used scoring systems, the prognostic value of procalcitonin was found to be quite high, similar to our findings [26] However, the procalcitonin cut-off value in this study was calculated as a low value like 0.5 ng/mL, and the number of patients evaluated for procalcitonin level in this study is low. Therefore, it seems necessary to conduct studies with larger patient samples on this subject. In a study conducted in 2023 in the literature, it was concluded that patients who have a procalcitonin level >2.25 ng/mL and fail to reduce procalcitonin levels below 60% of the initial value on the 7th day after treatment carry a high risk of death [27].

One of the strengths of the study is that it includes a large case series from a tertiary referral center hospital. The retrospective design and the inability to determine some parameters in certain cases due to data deficiencies are among the limitations of the study.

## 5. Conclusion

There is no scoring system that has been agreed upon to be the most successful in predicting local complications, necrosis, mortality, and disease severity in Acute Pancreatitis (AP). Especially in the early stages of AP, existing scoring systems or laboratory values alone are far from providing sufficient prognostic data. The use of inflammatory parameters in conjunction with scoring systems can contribute to diagnostic performance. The introduction of artificial intelligence technologies that can process much larger data in a short time and produce new systems may close the gap in this area.

## Figures and Tables

**Table:** Statistical analysis of the results of different scoring systems

	Cut-off	AUC(%95 Cl) (p value)	Sensitivite (95% Cl)	Spesifite (95% Cl)	PPV (95% Cl)	NPV (95% Cl)
Ranson score Severity Mortality Local Complications Necrosis	>4 >4 >2 >2	0.881 (84.0–91.5) (<0.001) 0.918 (88.2–94.6) (<0.001) 0.638 (57.2–70.1) (=0.001) 0.698 (63.0–76.1) (<0.001)	60.0 (26.2–87.8) 70.0 (34.8–93.3) 44.6 (31.3–58.5) 51.2 (34.8–67.6)	96.7 (94.0–98.4) 97.0 (94.5–98.6) 77.3 (70.3–83.4) 76.4 (69.1–82.7)	37.5 (21.4–57.0) 43.8 (26.7–62.5) 39.1 (30.0–48.9) 34.5 (25.8–44.3)	98.7 (97.2–99.4) 99.0 (97.4–99.6) 81.1 (77.0–84.6) 86.6 (82.3–90.0)
APACHE II score Severity Mortality	>7 >7	0.867 (80.1–91.7) (<0.001) 0.792 (71.9–85.4) (<0.001)	88.8 (51.8–99.7) 90.0 (55.5–99.7)	67.8 (59.4–75.5) 68.9 (59.7–75.7)	15.1(11.3–19.9) 16.7 (12.7–21.6)	99.0(93.7–99.8) 99.0 (93.7–99.8)
BISAP score Severity Mortality	>2 >2	0.884 (0.84–0.91) (<0.001) 0.829 (0.78–0.86) (<0.001)	70.0 (34.8–93.3) 63.6 (30.8–89.1)	93.1 (89.9–95.6) 93.2 (90.0–95.6)	23.3(14.7–34.9) 23.3(14.4–35.6)	99.1(97.6–99.6) 98.7(97.3–99.4)
mCTSI Severity Mortality Local Complications Necrosis	>2 >4 >2 >4	0.795 (73.7–84.5) (0.0002) 0.862 (81.1–90.4) (<0.001) 0.929 (88.8–95.9) (<0.001) 0.935 (89.5–96.3) (<0.001)	87.5 (47.3–99.7) 77.7 (40.0–97.2) 93.7 (84.8–98.3) 80.49(65.1–95.2)	64.4 (57.7–70.7) 82.5 (76.9–87.3) 83.5 (77.0–88.9) 93.55(89.0–96.6)	8.1(6.1–10.8) 15.2(10.3–22.0) 69.0 (61.0–75.9) 73.3 (60.9–82.9)	99.3(95.8–99.9) 98.9(96.4–99.7) 97.2 (93.0–98.9) 95.6 (92.1–97.6)

APACHE II; Acute Physiology and Chronic Health Enquiry, BISAP; Bedside Index Of Severity In Acute Pancreatitis, MCTSI; Modified Computed Tomography Severity Index, AUC; Area Under the Curve, PPV; Positive Predictive Value, NPV; Negative Predictive Value
